# Our New York Letter

**Published:** 1892-01

**Authors:** Wm. L. Russell

**Affiliations:** 151 East Fiftieth St.; New York


### OUR NEW YORK LETTER.

New York, November 16, 1891.

### ETIOLOGY OF CHANCROID.

At the last meeting of the Academy Section on genito-urinary diseases, the Chairman, Dr. R. W. Taylor, read a paper on the etiology of chancroid. He stated that his own researches, in connection with those of Dr. Bumstead, had overturned the dualistic doctrine of the origin of venereal sores. Chancroid was not caused by any specific virus, and Fournier was wrong in claiming that the presence of such a sore indicated positively that there had been contact with another chancroid. It could be produced by contact with any pus rich in pyogenic microbes, and bore the same relation to a mucous membrane that impetigo and ecthyma did to the skin. He had himself seen chancroid result in the male when the female organs were free from similar lesion. In one such instance he had discovered an ulcerating fissure of the cervix, which was intensely inflamed. He had also observed sores in all respects similar to chancroid, which had originated without sexual intercourse at all. Herpetic lesions might degenerate into ulcers. He had seen a case in which such ulcers had recurred at intervals for years, and although they had always been called chancroids by physicians, the patient was sure that they often appeared irrespective of intercourse. In another instance buboes resulted and auto-infection of the thighs, from ulcers due to herpes, at a time when the patient had not had intercourse for three months. Syphilitic subjects were more prone to chancroid, as their tissues were more susceptible.

Dr. P. A. Morrow thought that the cases in which chancroid resulted from infection with simple pus were exceptional; in the immense majority of cases chancroid was produced by chancroid or chancroidal bubo. Herpetic lesions might ulcerate, and the sores might resemble chancroid; the breaking down of tubercular lesions in syphilitic subjects might also cause similar sores, but the exceptions to the rule formulated by Fournier were so few as to be disregarded. Certain conditions of uncleanness and excess tended to foster the development of chancroid, but were not the actual causes.

Dr. G. E. Brewer would abolish the term chancroid altogether, as misleading. Chancres and herpes were produced by certain specific causes. It was not so with chancroid, which were simply septic sores, and should be so referred to.

Dr. Taylor said, in closing, that it had been proved experimentally that lesions similar to chancroid could be produced by inoculations with pus from impetigo and ecthyma. He thought, then, that these sores should be regarded as merely examples of wound infection.

### NITRATE OF SILVER IN GONORRHŒA.

At the same meeting Dr. Ramon Guiteras gave an account of his experience in treating gonorrhœa with injections of solutions of nitrate of silver of increasing strength. He gave a short history of the use of this remedy in the treatment of gonorrhœa, beginning fifteen or twenty years ago, when weak solutions of one-fifteenth of a grain to the ounce were used. Later the abortive treatment by the injection of strong solutions was practiced by some. Bumstead had used a drachm of a solution containing sixty grains to the ounce. This treatment was not successful as an abortive measure, however, and sometimes caused peri-urethritis. Nitrate of silver was not much used at present except in long continued cases when the deep urethra and bladder was effected. In these cases the deep injection of a few drops of a strong solution by means of a Keyes' or other suitable syringe was extremely useful. He had tried the remedy in acute cases and had had considerable success. His method consisted in the daily injection of a solution containing at first one grain to the ounce and increasing the strength up to fifteen grains to the ounce in some cases. In the majority, a solution containing eight or ten grains to the ounce was sufficient. Three to five injections produced a cure in some instances, and as the cases had not returned for further treatment, he believed the cure to be permanent. The only other treatment prescribed was the use of a solution of borax—a drachm to four ounces—as an injection to be used by the patient. His observations had led him to conclude that the use of nitrate of silver in gonorrhœa was not dangerous, but that the point of toleration was reached in most cases in a solution of eight grains to the ounce. A dry, irritable, congested condition of the mucous membrane remained afterwards, which required a mild astringent.

Dr. Brewer said that his experience in the treatment of gonorrhœa had produced three stages of feeling in him. The first had been a stage of dissatisfaction, when he had used internal medication; the next was one of enthusiasm, when he had treated a large number of cases with injections of solution of bichloride of mercury (see July number). He had now reached a stage of despair. He thought the nitrate of silver well worth trying, and he had already seen it do good in some cases.

Dr. Morrow's experience with nitrate of silver had not been so satisfactory as that of Dr. Guiteras. He had not used it in exactly the same way, however, and would adopt the plan suggested, at least tentatively.

Dr. Agramonte said that the success of nitrate of silver in the treatment of gonorrhœal ophthalmia was a strong argument for its adoption as a remedy for gonorrhœal urethritis. It had been proved experimentally that a solution of five grains to the ounce would destroy the gonococci instantly, and he thought there was no reason for employing solution of any greater strength.

### THE KEELEY "CURE" FOR DRUNKENNESS.

The Keeley "cure" for drunkennes and the opium habit has come among us. A branch establishment has been opened at White Plains, a few miles away, and there hundreds of unfortunates are flocking daily. Dr. Keeley, the discoverer of the "cure," lives in Dwight, Ill., and has advertised for several years in a small pamphlet, setting forth his long experience, and the wonderful efficacy of the bichloride of gold, prepared and used properly by him only. It is only recently, however, that he has attracted general attention. Several "cured" drunkards, some of them quite prominent people, have announced the fact of their recovery from the disease of drunkenness, as it is termed by Dr. Keeley in the public press, and the result is that a large increase in Dr. Keeley's income has occurred. It is stated that as many as six hundred patients have been observed in line before his office waiting to receive a hypodermic injection. Four injections are given daily, medicine being taken by the stomach every two hours during the interval. No restrictions are placed on the patient while under treatment, as the remedy is said to remove all desire for stimulants. It is claimed that 95 per cent. are cured.

The newspapers have devoted considerable attention to the propriety of concealing the nature of the remedy, some taking one view and some another. It is stated that Dr. Keeley keeps his secret because of Koch's experience, and that if the medical fraternity will acknowledge the justice of his claims beforehand he will publish every fact connected with it. It must be said, however, that the prevailing opinion expressed by the newspapers is that the actuating motive is the desire for gain rather than the good of humanity; a silent tribute thus being paid to the time-honored rule of the profession regarding inventions and discoveries.

The remedy is announced by Dr. Keeley to be a double chloride of gold, but its exact nature and the manner of using it have not been explained, and all our knowledge of its virtues is derived from Dr. Keeley's claims and the statements of those who claim to have been cured. Among the more prominent of these was Mr. C. F. Mines, known in literature as "Felix Oldboy," who gave a forcible account of his experience in the *North American Review*. He expressed his satisfaction of a cure, which had then lasted for two years. His recent death from the effects of a prolonged spree can hardly be regarded as favorable to Dr. Keeley's claims. I have also heard of another case who was discharged "cured," and four days later started out as before to drink himself dead drunk.

As the remedy has not yet been scientifically investigated, a judgment on its merits cannot be pronounced. It is certain, however, that the history of medicine does not warrant us in expecting great benefits to humanity to accrue from remedies first announced on tinted advertisements sent out to attract patients to the alleged discoverer.

The remarks of Oliver Wendell Holmes at the opening of the Academy about a year ago recur to me now with renewed significance. "Nowhere is such a defence more needed than in the science and arts which deal with the health of the community. The public is so ready, so eager to be deceived, and the traders in deception are so willing, so hungry to deceive those who will listen to them, that it needs a solid wall of resistance, a close phalanx of men of recognized sense, knowledge and character to stand against them."

WM. L. RUSSELL.

151 *East Fiftieth St.*

---

TREATMENT OF HEPATIC COLIC BY OLIVE OIL.—Dr. Willemin presents in *Bulletin Generale de Therapeutique* an account of nineteen cases of hepatic colic occurring in the practice of others, which have been successfully treated by the use of olive oil, and nine cases occurring in his own practice treated by the same agent. In all these cases there was a cessation of pain soon after administering the oil. The usual quantity given was six ounces of the pure oil, repeating the dose until all pain had disappeared. Nausea and vomiting promptly ceased, and the icterus gave way before the use of the remedy. The fever sometimes existed a longer time. If a second attack followed, another dose of the oil was given with the same happy results. The oil sometimes gave relief to pain when light foods and opiates could not be tolerated by the stomach. Many months sometimes elapsed before the attacks returned, if at all. The author does not believe it possible to relieve the sufferings of hepatic colic by suggestion alone, but maintains that something more is necessary to relieve these horrible pains. Olive oil seems to act as a solvent of the biliary concretions, rendering them more difficult of detection after they have been passed.—*Medical Age.*

